# Correction: Short Report: Early genomic detection of SARS-CoV-2 P.1 variant in Northeast Brazil

**DOI:** 10.1371/journal.pntd.0011024

**Published:** 2022-12-30

**Authors:** Stephane Tosta, Marta Giovanetti, Vanessa Brandão Nardy, Luciana Reboredo de Oliveira da Silva, Marcela Kelly Astete Gómez, Jaqueline Gomes Lima, Cristiane Wanderley Cardoso, Tarcisio Oliveira Silva, Marcia São Pedro Leal de Souza, Pedro Henrique Presta Dias, Vagner Fonseca, Tulio de Oliveira, José Lourenço, Luiz Carlos Junior Alcantara, Felicidade Pereira, Arabela Leal

There is an error in the name of one of the authors. The name Pedro Henrique Presta Dia should be listed as Pedro Henrique Presta Dias.

## References

[1] Tosta S, Giovanetti M, Brandão Nardy V, Reboredo de Oliveira da Silva L, Kelly Astete Gómez M, Gomes Lima J, et al. (2021) Short Report: Early genomic detection of SARS-CoV-2 P.1 variant in Northeast Brazil. PLoS Negl Trop Dis 15(7): e0009591. doi: 10.1371/journal.pntd.0009591 34280196 PMC8321350

