# Supplementary figures and images for: Short Report: Early genomic detection of SARS-CoV-2 P.1 variant in Northeast Brazil
Source: PLoS Negl Trop Dis. 2021 Jul 19;15(7):e0009591. doi: 10.1371/journal.pntd.0009591 (PMC8321350; doi:10.1371/journal.pntd.0009591)

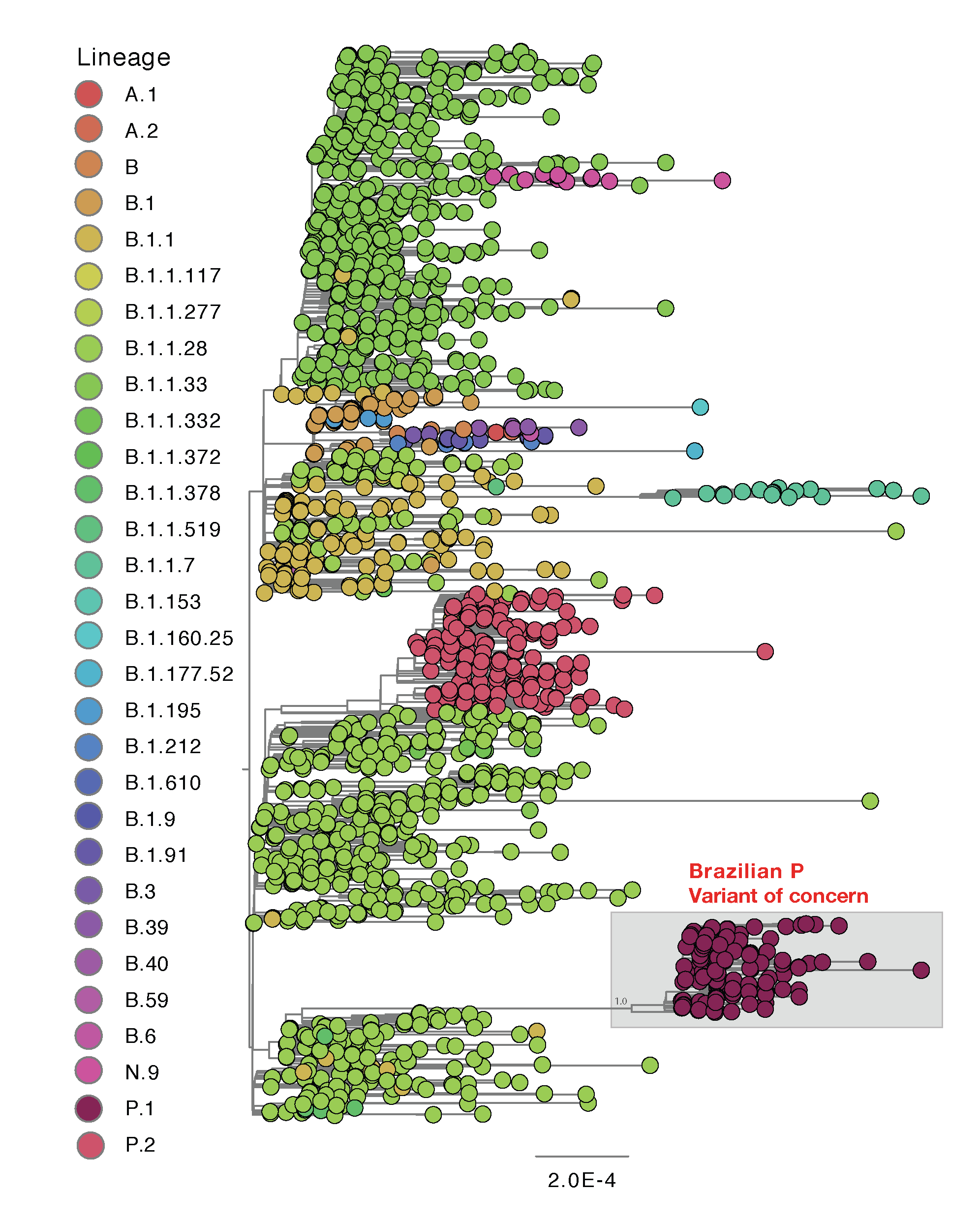

Supplement: S1 Fig — (TIF) [file pntd.0009591.s002.tif]
