# Supplementary material for: Short Report: Early genomic detection of SARS-CoV-2 P.1 variant in Northeast Brazil
Source: PLoS Negl Trop Dis. 2021 Jul 19;15(7):e0009591. doi: 10.1371/journal.pntd.0009591 (PMC8321350; doi:10.1371/journal.pntd.0009591)
Supplement: Attachment — Submitted filename: Point_to_point.docx [file pntd.0009591.s003.docx]

Dear Editor,

We are pleased that the editorial assessment of our manuscript was largely favourable. We have addressed all comments, and we believe that the resulting manuscript is much improved. We hope you will consider the revision to be suitable for publication.

**Reviewing: 1**
Tosta et.al. present the manuscript entitled “Early genomic detection of SARS-CoV-2 P.1 variant in Northeast Brazil” described a pilot study in the northern region of brazil to recover the 11 SARS-COV2 genomes contain P.1 variant, which is of the active public health concern strain. As P1 variants is the important for public health on the active COVID19 pandemic right now, the manuscript will be interested in researchers of the closely related filed. However, the manuscript needs to be improved, especially in the method section. The inconsistency between method and result needs to be clarified.

***Reply:*** *We thank the reviewer for his positive comment. We have addressed all comments in the detailed point to point revision (see below).*

**Comment 1:** The P.1 variant need to be defined in detail at first in the Author summary section

***Reply****:* *We agree with the reviewer and we appreciate the suggestion. Changed accordingly.*

**Comment 2:** Please provide version of the software used in the study: Guppy, qcat, MAFFT, IQ_TREE

***Reply****:* *We appreciate the reviewer comment and we have made the necessary changes.*

**Comment 3:** As Phylogenetic tree analysis is very important result of the study, I recommend the author give the reader more details how to construct the tree such as what is the parameter used, what is the evolutionary model used. Boot strapping is highly recommended to perform and report as confident of the tree result.

***Reply****:* *Improved accordingly.*

**Comment 4:** It is very inconsistency in the method and result! The authors mentioned that they use Oxford Nanopore sequencing of PCR product from ARCTIC primer set, but the first paragraph of the result mentioned that Ion torrent sequencer was used. The authors need to clarify experimental procedures in a better detail.

***Reply****:* *The reviewer is right,* we *really apologize for the mistake and for the confusion this might have caused. We have now deeply edited this whole section and provided a proper description of the methods used.*

**Comment 5:** Figure 1A need to be improved. The annotation of different clades based on known linages need to be reported on the figure. Readers will not gain any knowledge from the figure without the annotation of leakages. By using “Others” is meaningless. Standard tree file format such as newick format need to be shared as a supplementary for the readers.

***Reply:*** *We respectfully disagree with the reviewer. The objective of the present article is describing the early detection of the SARS-CoV-2 P.1 variant of concern (VOC) in the Northeast of Brazil (Bahia state). This is why a special focus have been done to this VOC into the tree. Despite this we agree with the reviewer that a detailed annotated tree need to be added as a supplementary figure (****Figure S1****). Standard tree file is also now available into the GitHub Public repository at: https://github.com/genomicsurveillance/P1_Northeast_Brazil*

**Comment 6:** The label on y-axis of Figure 1D is missing.

***Reply:*** *The reviewer is correct. We have made the necessary changes.*

**Comment 7:** Raw sequence data need to be deposited and shared for the research community in a public database such as NCBI SRA database.

***Reply:*** *We appreciate the reviewer comment and we have now deposited the raw data into the NCBI SRA database (Project Number: PRJNA729484: Short Report: Early genomic detection of SARS-CoV-2 P.1 variant in Northeast Brazil).*

**Comment 8:** For reproducible and transparency of the result, I recommended the authors share the computational notebook or chunk of code used in the study.

***Reply:*** *R code to reproduce the analyses and figures presented in this paper are available at https://github.com/genomicsurveillance/P1_Northeast_Brazil*

**Reviewing: 2**

The manuscript “Early genomic detection of SARS-CoV-2 P.1 variant in Northeast Brazil” provides a brief report resulting from the analyses of 11 viral genomes. While the dataset is limited, it comes from an epidemiologically interesting region at an important time in the dispersal of the P.1 variant. As such, the manuscript provides information that will be of general interest. The message is simple and clearly presented by the authors. Some minor comments for the authors consideration follow.

**Comment 1:** Ln 78. The authors state that eleven suspected P1 infections resulted from the massive screening program. It is a little unclear exactly what this means. Were only 11 positive samples detected through this screening (I suspect this is not what is meant)? It might be clearer if the number of tests conducted, and the number of positive PCR tests were provided. If more than 11 samples were positive, how were these particular samples selected?

***Reply****:* *We thank the reviewer for the comment. At the time when this surveillance was done, only 11 positive samples were available for screening and investigation.*

**Comment 2:** Ln 129. What was the rationale for using two PCR assays on each sample?

***Reply:*** *We appreciate the reviewer comment.* *The unique rationale was based on the availability of the assay supplied by the Brazilian Ministry of Health which currently are the: i) Allplex™ 2019-nCoV Assay (Seegene) and the ii) Charité (Biomanguinhos).*

**Comment 3:** Ln 136. Did all four family members travel, or was there evidence of within household spread?

***Reply:*** *We appreciate the reviewer comment. All family members reported travel history to/from the Amazonas state.*

**Comment 4:** It would be of interest to know the spectrum of disease experienced by these individuals.

***Reply*** *We appreciate the reviewer comment and we have now provided additionally details (****Table 1****).*

**Editorial suggestions**

**Comment 1:** Ln 133. “generated where assigned” should be “generated were assigned”

***Reply****:* *Done.*

**Comment 2:** Ln 135. “recently record travel” should be “recently recorded travel”

***Reply****:* *Done.*

**Comment 3:** Ln 136. “back into the” should be “back to the”

***Reply****:* *Done.*

**Comment 4:** Ln 136-137. “and the other others had” should be “and the others had”

***Reply****:* *Done.*
